# A comparison of exposure of Glottis using the Airtraq or the Macintosh Laryngoscope in Potentially difficult airway management: A self-controlled clinical trial

**DOI:** 10.12669/pjms.344.14411

**Published:** 2018

**Authors:** Ji-Ming Wang, Er-Li Ma, Yun-Xia Zuo, Jing Lin, Peng Liang, Xiao-Qiang Li

**Affiliations:** 1 Ji-Ming Wang, MD. Department of Anesthesiology, Shenzhen Bao’an Shajing People’s Hospital, Guangzhou Medical University, Shenzhen 518104, Guangdong, China; 2 Er- Li Ma, MD. Department of Anesthesiology, West China Hospital, Sichuan University, Chengdu 610041, Sichuan, China; 3 Yun-Xia Zuo, MD, PhD. Department of Anesthesiology, West China Hospital, Sichuan University, Chengdu 610041, Sichuan, China; 4 Jing Lin, MD. Department of Anesthesiology, West China Hospital, Sichuan University, Chengdu 610041, Sichuan, China; 5 Peng Liang, MD. Department of Anesthesiology, West China Hospital, Sichuan University, Chengdu 610041, Sichuan, China; 6 Xiao-Qiang Li, MD. Department of Anesthesiology, West China Hospital, Sichuan University, Chengdu 610041, Sichuan, China

**Keywords:** Airtraq laryngoscopy, Potential difficult tracheal intubation, Glottis exposure, Macintosh laryngoscopy

## Abstract

**Objective:**

To compare glottis exposure of the same patients with potentially difficult tracheal intubation (PDTI) subjected to Airtraq laryngoscopy and Macintosh laryngoscopy under consciousness and topical anesthesia.

**Methods:**

A total of 147 PDTI patients with American Society of Anesthesiologists (ASA) I-III were subjected to Airtraq and Macintosh laryngoscopy performed by experienced anesthesiologists under consciousness and topical anesthesia.

**Results:**

All patients were successfully intubated. Among them, three patients were intubated with fiberoptic bronchoscopy, 13 with Macintosh laryngoscopy and 131 with Airtraq laryngoscopy. Of the patients with Cormack and Lehance (C&L) Grade-I glottic view, 88 were subjected to Airtraq laryngoscopy and five to Macintosh laryngoscopy; Of the patients with C&L Grade-II glottic view, 56 were subjected to Airtraq laryngoscopy and 21 to Macintosh bronchoscopy; Of the patients with C&L Grade-III glottic view, three were subjected to Airtraq laryngoscopy and 112 to Macintosh bronchoscopy; Of the patients with C&L Grade-IV glottic view, none was subjected to Airtraq laryngoscopy and 9 to Macintosh laryngoscopy.

**Conclusions:**

Airtraq laryngoscopy could significantly improve the glottis exposure and reduce the difficulty of intubation for patients with potentially tracheal intubation compared to the traditional Macintosh laryngoscopy.

## INTRODUCTION

Management of anticipated difficult airway is a major challenge for the anesthesiologists. The failure to successfully intubate the trachea and secure the airway for patients remains a leading cause of morbidity and mortality in anesthesia practice.^1^-^3^ The absence of method that reliably predicts the existence of a difficult airway means that many difficult intubations are not known until induction of anesthesia.^4^,^5^ A wide variety of alternative airway devices and tools have been developed and, in part, successfully implemented in difficult airway management algorithms.^6^ Airtraq is a new intubation device that was initially designed to provide a view of the glottis without alignment of the oral, pharyngeal and tracheal axes and has developed to facilitate tracheal intubation in patients with normal airways.^7^ The blade of the Airtraq consists of two side-by-side channels, of which, one is used to guide the endotracheal tube and the other with lens and prisms is used to acquire a visually controlled endotracheal intubation.

In many studies, Airtraq provided superior endotracheal intubation conditions and enabled higher success rate in laryngoscopy than conventional laryngoscopy, particularly in routine airway^7^ or morbidly obese patients.^8^ However, very few studies have applied Airtraq in potentially tracheal intubation and especially compared Airtraq laryngoscopy with Macintosh laryngoscopy for glottis exposure in the same patients.

It has been speculated that Airtraq intubation may be easier than Macintosh laryngoscopy intubation when utilized for patients with potentially tracheal intubations. Therefore, it appears worthwhile to assess the potential advantages of Airtraq endotracheal intubation in patients with potentially difficult tracheal intubation (PDTI). Therefore, we conducted a prospective self-controlled study to compare the glottis exposure of the same PDTI patients under the condition of conscious anesthesia by using Macintosh laryngoscopy and Airtraq laryngoscopy sequentially operated by the same experienced anesthesiologist. The hypothesis of this study was that the difficulty of intubation should be significantly improved by Airtraq laryngoscopy than traditional Macintosh laryngoscopy.

## METHODS

The study was approved by the Institutional Ethics Committees of West China Hospital and performed by the participating hospitals that registered at the clinical trial database with registration number of ChiCTR-TRC-11001418. A written and informed consent was obtained from each participant. Patients at age of 18-65 years old with American Society of Anesthesiologists (ASA) classification scores I-III and potentially difficult ventilation and difficult intubation requiring tracheal intubation for their elective surgeries were enrolled. The inclusion criteria were as follows: 1) Patients with oral, upper airway and trachea tumor or neoplasm; 2) Patients with tracheal compression by cervical neoplasms or mass; 3) Patients with tracheal compression by anterior mediastinal tumors; 4) Patients with tracheal deviation or stenosis caused by neck trauma, burn, surgical procedures and radiotherapy; 5) Patients with body mass index (BMI) ≥30, Mallampati score III-IV and thyromental distance <6.0 cm^9^ and 6) Patients with obstructive sleep apnea (OSA) with apnea-hypopnea index (AHI) ≥20.

The general information and detailed airway assessments of the enrolled patients were documented preoperatively. Routine monitoring was established including electrocardiography, blood pressure, pulse oximetry (SpO2) and capnography. For each enrolled patient, 0.5 mg atropine was administered intravenously to keep the airway dry and ephedrine was used to prepare the nostrils in case there was a need for nasal intubation. In addition, preoxygenation employing the bag mask valve prior to intubation was applied to all patients, and the level of arterial oxygen saturation was real-timely monitored. Vocal cord exposure was evaluated in conscious state with light sedation and topical anesthesia. Briefly, 2% lignocaine or 1% tetracaine was topically administered to the airway with or without maximum 2 mg midazolam (0.5 mg each time) and maximum 100 µg fentanyl (20 µg each time) given intravenously based on the assessment of the care team. Each patient was first exposed with traditional Macintosh laryngoscopy and followed by Airtraq laryngoscopy.

**Fig.1:**
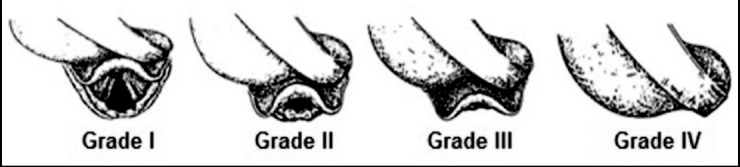
Cormark and Lehance (C&L) grading system.^23^

The attending anesthesiologist in charge of the case assessed and rated the difficulty of vocal cords exposure (recorded by the investigator) according to Cormack and Lehance (C&L) classification^10^ (Fig.1). If C&L grade ≥3, video-assisted Airtraq laryngoscopy was primarily utilized. If C&L grade equaled to or were greater than III, intubation was conducted with adjunct of a fiberoptic bronchoscope (FOB).^11^

The primary outcome was the glottis view rating according to the C&L classification scale and the secondary outcome was successful establishment of an endotracheal airway. The standard of successful tracheal intubation was the tracheal tube placed through the glottis as confirmed visually by the anesthesiologist if the endotracheal tube (ETT) was not visualized passing through the vocal cords, the intubation attempt was not considered complete until the ETT was connected to the anesthetic circuit and evidence showed the presence of carbon dioxide in the expiration.

Based on the assumption that the success rate of endotracheal intubation in the field should be 95% for Macintosh laryngoscopy and 80% for Airtraq laryngoscopy after basic training as suggested in literature^12^,^13^, the sample size needed to detect a significant difference between groups at *p* < 0.05 with a drop out event for any reason being 20% was calculated to be n =140.

Statistical analyses of quantitative data were performed by using SPSS17. Data were presented as mean ± SD or median with 95% confidence interval for non-Gaussian variables. Normal distribution of all linear data was examined by Kolmogorov-Smirnov test. Two-sided differences among groups at the primary and the secondary end points were examined. Nonparametric data were compared using rank sum test. Comparison of percentages was performed using either a Chi-square or Fisher exact test. Parametric data between the two groups were compared using the Student *t*-test.

## RESULTS

A total of 147 patients were enrolled in the study. The demographic or baseline airway parameters of these patients including BMI, mouth opening, neck circumference, Mallampati classification and thyromental distance, which were used to assess the potential difficulties of airway intubation are listed in Table-I. Among them, 128 patients had Mallampati classification grade≥3 preoperatively.

**Table-I:** Demographic or baseline airway parameters and intubation.

Age (yr)	37.7±10.6
Gender (M/F)	126/21
Body mass index(BMI)	27.3±3.9
ASA	
I	72
II	55
III	20
Mallampati classification	
I	1
II	18
III	113
IV	15
Mouth opening (cm)	4.3±0.9
Neck circumference (cm)	41.9±3.6
Thyromental distance (cm)	7.3±1.1
AHI	57.1±19.8
ML intubation	13 (8.8%)
Airtraq intubation	131 (89.1%)
FOB intubation	3 (2.1%)

AHI: Apnea hypopnea index, ML: Macintosh laryngoscopy, FOB: Fiberoptic bronchoscopy. Data are reported as mean ± SD or number (%).

**Fig. 2:**
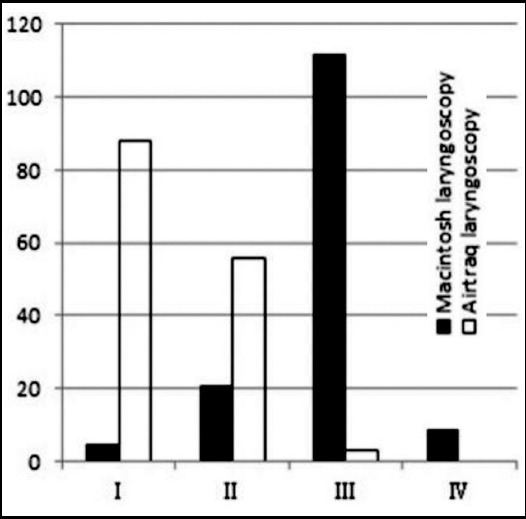
Glottis view comparing the Cormark and Lehane grade of patients subjected to Macintosh laryngoscopy and Airtraq laryngoscopy.

Patients with snoring disease and patients with neck masses were also included in the study (Table-II). All patients were successfully intubated. Among them, 3 (2.1%) patients were intubated with fiberoptic bronchoscopy, 13 (8.8%) with Macintosh laryngoscopy and 131 (89.1%) with Airtraq laryngoscopy (Table-I). Of the patients with C&L Grade-I glottic view, 88 were subjected to Airtraq laryngoscopy and five to Macintosh laryngoscopy; Of the patients with C&L Grade-II glottic view, 56 were subjected to Airtraq laryngoscopy and 21 to Macintosh bronchoscopy; Of the patients with C&L Grade-III glottic view, three were subjected to Airtraq laryngoscopy and 112 to Macintosh bronchoscopy; Of the patients with C&L Grade-IV glottic view, none was subjected to Airtraq laryngoscopy and 9 to Macintosh laryngoscopy (Table-III, Fig.2). The C&L grades were significantly different between patients subjected to Airtraq and Macintosh laryngoscopy (*p*<0.001).

**Table-II:** Classification of patients with potentially tracheal intubation.

Types of patients	OSA	Neck mass	Other patients
Number	122	23	2

Data are reported as number. OSA: Obstructive sleep apnea

**Table-III:** Summary of Cormack and Lehane grade of same patients subjected to Macintosh laryngoscopy and Airtaq laryngoscopy based on Glottic view.

C&L	ML	*p*<0.001	I	II		III			IV		
	
	AL		I	I	II	I	II	III	I	II	III
Number			5	19	2	59	52	1	5	2	2
(%)			100	90.5	9.5	52.7	46.4	0.9	55.6	22.2	22.2

Data are reported as number (%). Cormack and Lehane grade: C&L grade. Macintosh laryngoscopy: ML, Airtaq laryngoscopy: AL. The C&L grades were significantly different between patients subjected to Airtraq and Macintosh laryngoscopy (P<0.05).

The figure shows that the Airtraq laryngoscopy shifted the C&L grading for glottic exposure from higher grade to the lower grade. The majority were classified as graded I and II with Airtraq larygoscopy but III or IV with Macintosh laryngoscopy.

## DISCUSSION

It has been recommended that after initial manikin assessment and comparison of tracheal intubation using Airtraq or Macintosh laryngoscopes in routine airway management, all new airway devices should be compared in a randomized controlled trial against the current gold standard.^14^,^15^ The performance of Airtraq laryngoscopy has been assessed previously in manikins and in the normal airway. When used by anesthetists, relatively inexperienced medical personnel^16^ and novice users^7^,^17^, Airtraq laryngoscopy has demonstrated potential advantages in both easy and simulated difficult laryngoscopy scenarios. The curved laryngoscope blade described by Macintosh in 1943 remains the most popular device used to facilitate orotracheal intubation notwithstanding recent developments in airway device technologies, and therefore constitutes the gold standard.^7^ We therefore wished to compare the utility of the Airtraq to Macintosh laryngoscope in patients with potentially tracheal intubation.

All intubations in this study were performed by four experienced anesthetists. Each of them had performed >500 intubations using Macintosh laryngoscope, and at least 100 intubations with Airtraq in manikins and 100 intubations with Airtraq in patients prior to this study. More than 100 patients with potentially tracheal intubation were subjected to glottis exposure with Macintosh laryngoscope and Airtraq laryngoscope sequentially. Our study demonstrated that compared with Macintosh laryngoscope, Airtraq provides comparable or superior intubating conditions in patients with potentially tracheal intubation. A total of 147 patients enrolled in this study including those with snoring, neck mass, and other diseases (Table-II). Among them, 128 patients had Mallampati classification grade≥3. The successful rate of intubation was 89.1% for patients subjected to Airtraq laryngoscopy, significantly higher than that of 8.8% for patients subjected to Macintosh laryngoscopy, indicating that Airtraq did reduce intubation difficulty. (Table-I).

The C&L grading system, although originally designed to compare glottic views of Macintosh laryngoscopy, provided a useful comparison of the direct and indirect laryngoscopic views. One hundred forty four patients exposed with Airtraq laryngoscopy had C&L Grade I or II glottic view, while only 26 patients exposed with Macintosh laryngoscopy had C&L Grade I or II glottic view. With Airtraq, C&L Grade-IV glottic view of patients subjected to Macintosh laryngoscopy changed to C&L Grades I or II glottic view, of which, the grade of 55.6% patients improved to Grade-I and that of 22.2% patients improved to Grade-II. With airtraq laryngoscopy, patients with C&L Grade-III using Macintosh laryngoscopy became Grade I or II, of which, the grade of 52.7% patients improved to I and that of 46.4% patients improved to II. However, one patient with C&L Grade-III using Macintosh laryngoscopy did not improve with Airtraq (Table-III) and some patients required additional maneuvers to improve glottic exposure with fiberoptic bronchoscopy. This might occur when there was an oral lesion. Airtraq is one of the relatively inexpensive and widely used video-laryngoscopes in China.^6^,^18^ Airtraq adopted in the experiment attributed to less haemodynamic stimulation following tracheal intubation in comparison with Macintosh laryngoscope.^7^,^19^,^20^ This finding probably reflects the fact that Airtraq provides a view of glottis without the need to align the oral, pharyngeal and tracheal axes, and therefore requires less force during laryngoscopy. With Airtraq, 121 patients with C&L Grades III and IV using Macintosh laryngoscopy became Grade I or II (Fig.2). The rate of patients with difficulties in vocal cords exposure under Macintosh laryngoscope (C&L Grades III-IV) was reduced by 97.5% in the study. Airtraq offers significantly better views of the glottis compared with Macintosh (Table-III) in our study, which was consistent with previous report.^8^,^19^,^21^

### Limitations of the study

The majority of recruited patients were men who suffered from OSA. Therefore the proportion of men and women was not coordinated. This might suggest that male itself is a risk factor in the potential difficult airway.^22^ Further studies in the clinical context, particularly predicting subglottic difficult intubation scenarios, are necessary to confirm and extend this initial positive finding.

## CONCLUSION

Our results showed that Airtraq laryngoscopy could significantly improve the glottis exposure and reduce the difficulty of intubation for patients with potentially tracheal intubation compared to the traditional Macintosh laryngoscopy.

### Authors’ Contribution

**YXZ** was responsible for the project design and guidance in Department of Anesthesiology, West China Hospital, Sichuan University.

**ELM, PL and XQL** are responsible for project implementation.

**JMW and JL** are responsible for the data collection and follow up visits.

**JMW, ELM and YXZ** analyzed the whole data and prepared for the manuscript.
